# Acquired A amyloidosis from injection drug use presenting with atraumatic splenic rupture in a hospitalized patient: a case report

**DOI:** 10.1186/1752-1947-5-29

**Published:** 2011-01-24

**Authors:** Garrett R Roll, Andrew Y Lee, Kayvan Royaie, Brendan Visser, Douglas K Hanks, Margaret M Knudson, Frederick J Roll

**Affiliations:** 1 Department of Surgery, University of California San Francisco, San Francisco, USA; 2 Department of Surgery, Stanford University, Stanford, California, USA; 3 Department of Pathology, San Francisco General Hospital, San Francisco, USA; 4 Department of Surgery, San Francisco General Hospital, San Francisco, USA; 5 Department of Medicine, San Francisco General Hospital, San Francisco, USA

## Abstract

**Introduction:**

Little is known about splenic rupture in patients who develop systemic acquired A amyloidosis. This is the first report of a case of atraumatic splenic rupture in a patient with acquired A amyloidosis from chronic injection drug use.

**Case presentation:**

A 58-year-old Caucasian man with a long history of injection drug use, hospitalized for infective endocarditis, experienced atraumatic splenic rupture and underwent splenectomy. Histopathological and microbiological analyses of the splenic tissue were consistent with systemic acquired A amyloidosis, most likely from injection drug use, that led to splenic rupture without any recognized trauma or evidence of bacterial embolization to the spleen.

**Conclusion:**

In patients with chronic inflammatory conditions, including the use of injection drugs, who experience acute onset of left upper quadrant pain, the diagnosis of atraumatic splenic rupture must be considered.

## Introduction

Atraumatic splenic rupture (ASR) can be caused by neoplastic diseases, hematological disorders, infection and chronic inflammatory states [1]. Patients who are hospitalized rarely experience ASR, which carries a mortality of approximately 12% [1]. ASR from amyloidosis has been documented previously in three case reports, but no patients with chronic injection drug use as the etiological factor in the development of systemic acquired A (AA) amyloidosis have been described [2-5]. We report the case of a hospitalized patient who experienced atraumatic splenic rupture from acquired systemic AA amyloidosis, most likely resulting from chronic injection drug use.

## Case presentation

A 58-year-old Caucasian man with an extensive history of injection drug use was hospitalized with a diagnosis of infective endocarditis (IE); eight months earlier, he had experienced an episode of IE that was treated surgically with a bioprosthetic valve. Blood cultures taken during the current admission revealed methicillin-resistant *Staphylococcus aureus *infection. Transesophageal echocardiography revealed a 2 cm linear vegetation on the prosthetic aortic valve and a possible ring abscess without evidence of aortic insufficiency. Therapy with intravenous ciprofloxacin, gentamicin and levofloxacin was initiated.

**Figure 1:**
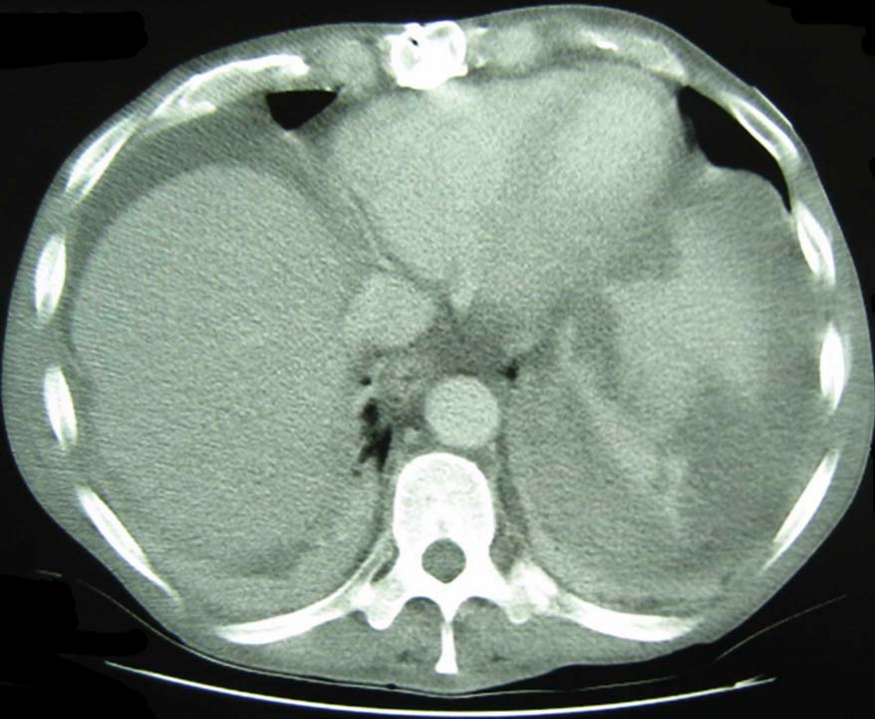
**Computed tomography (CT) scan**. The CT scan demonstrates hemoperitoneum and a grade IV splenic laceration in our patient, who had no history of trauma.

On the third day of hospitalization, our patient experienced an acute onset of left upper quadrant abdominal pain. No history of recent trauma could be elicited. Our patient's vital signs were as follows: temperature 40°C, blood pressure 85/48 mmHg, heart rate 104 beats/minute, respiratory rate of 20 breaths/minute, and an oxygen saturation of 99% breathing room air. On physical examination our patient was found to be in mild distress and diaphoretic with dry mucous membranes. The skin of the upper and lower extremities was indurated at sites of frequent subcutaneous injections, but without erythema, exudate or abscess. No petechiae of the skin or nail beds were identified. A cardiac examination revealed a III/VI crescendo/de-crescendo systolic ejection murmur radiating to the apex and to the carotid arteries. An abdominal examination revealed a distended abdomen and tenderness to palpation in the left upper quadrant. A computed tomography (CT) scan of the abdomen and pelvis revealed an enlarged and fractured spleen with surrounding hematoma, but no evidence of liver laceration, infarct or intra-abdominal abscess (Figure 1).

**Figure 2:**
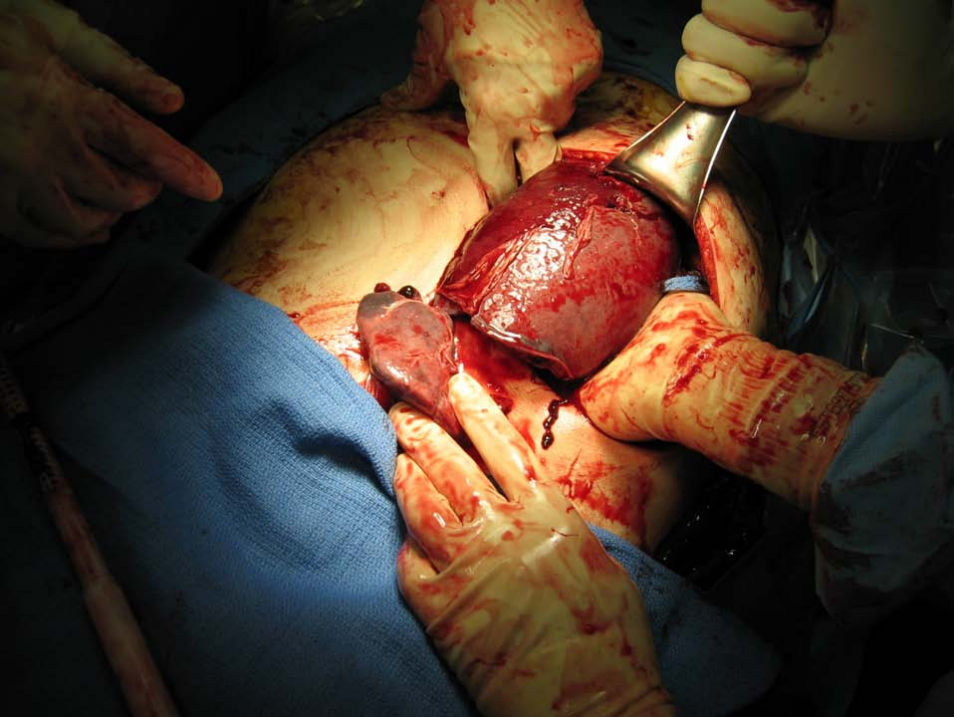
**Initial view of the gross spleen**. The enlarged and fractured spleen as seen through a left subcostal incision.

Our patient underwent an emergency splenectomy. A subcostal incision revealed approximately 2L of blood inside the abdominal cavity, an enlarged spleen with grade IV disruption (Figure 2), and an aneurysmal splenic artery. The spleen was freed at its attachments and the arteries and veins at the hilum were ligated. The aneurysmal splenic artery was dissected medially for sufficient proximal exposure and was then ligated. Abdominal exploration revealed no further gross pathological findings. A histopathological evaluation of other organs for evidence of amyloidosis was not performed. Our patient's early post-operative recovery was uneventful. However, he later died from complications of endocarditis and ring abscess.

**Figure 3:**
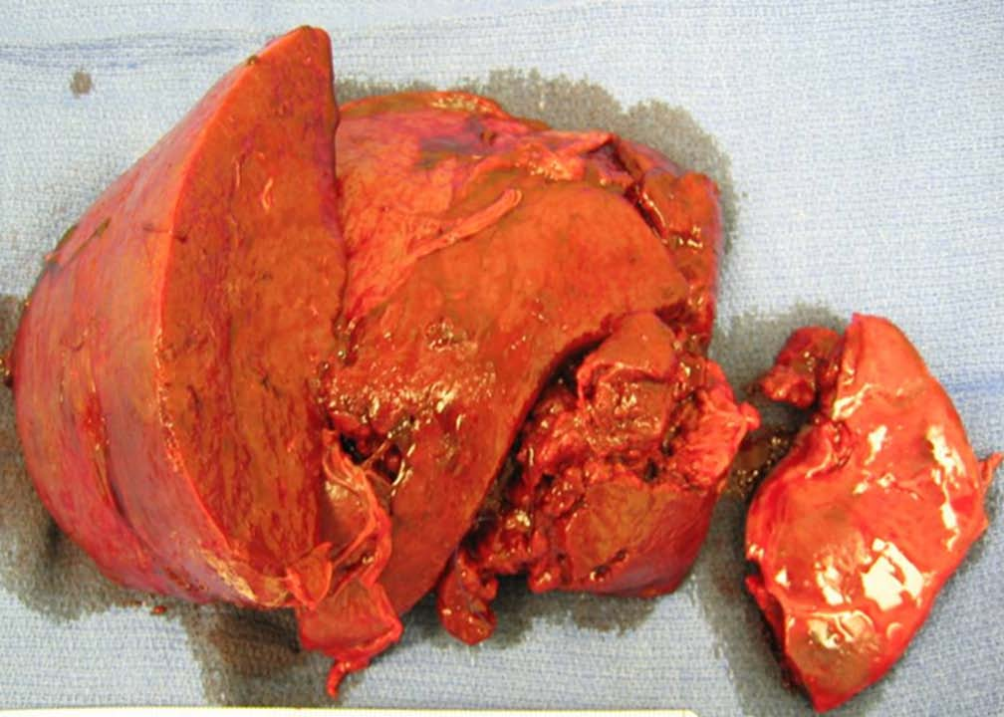
**Gross spleen**. The grossly enlarged spleen cut along the short axis with no evidence of abscess formation.

**Figure 4:**
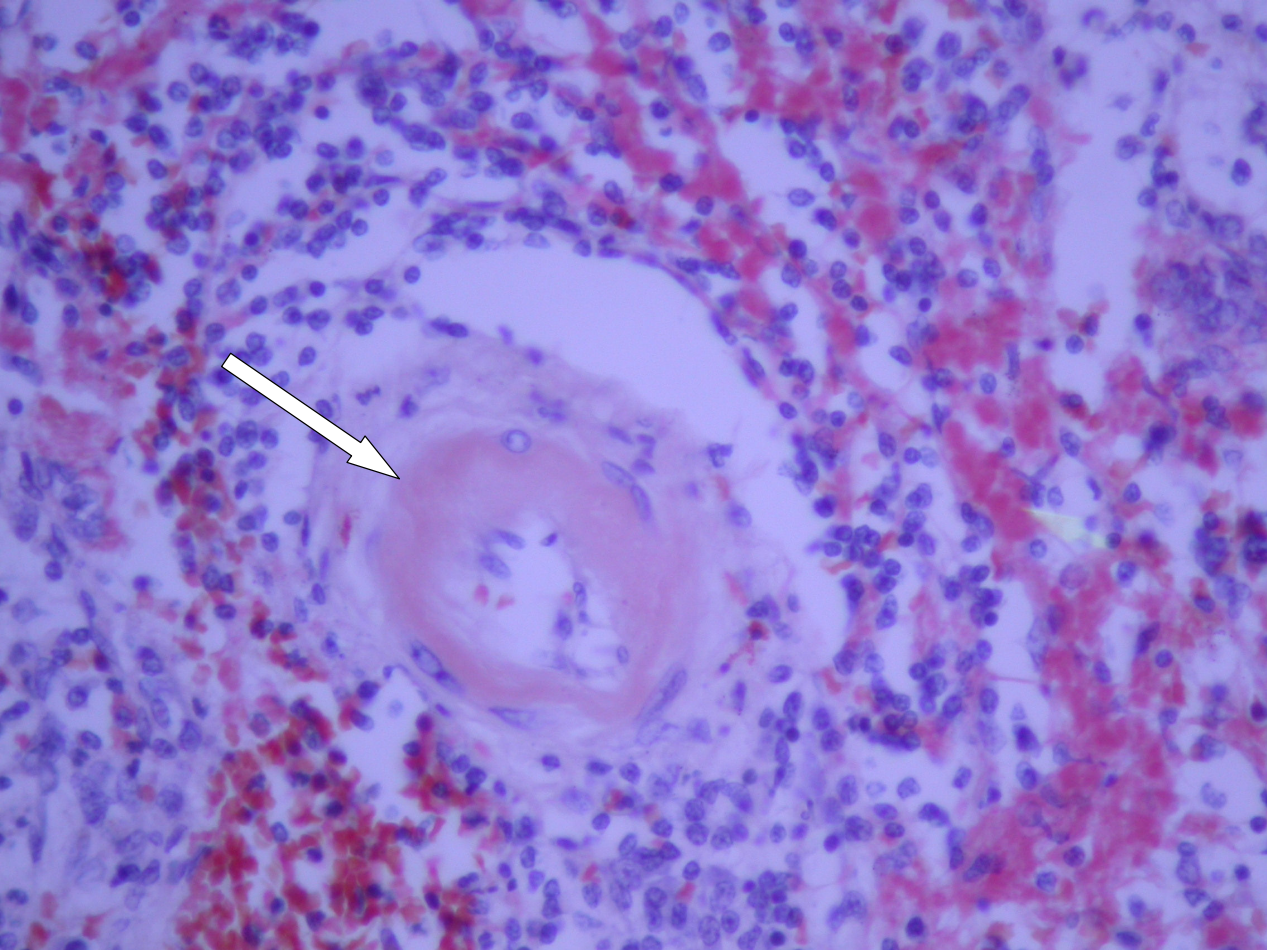
**Hematoxylin and eosin staining results**. Hematoxylin and eosin stain of splenic tissue at 40 × magnification showing amyloid around a blood vessel (arrow).

**Figure 5:**
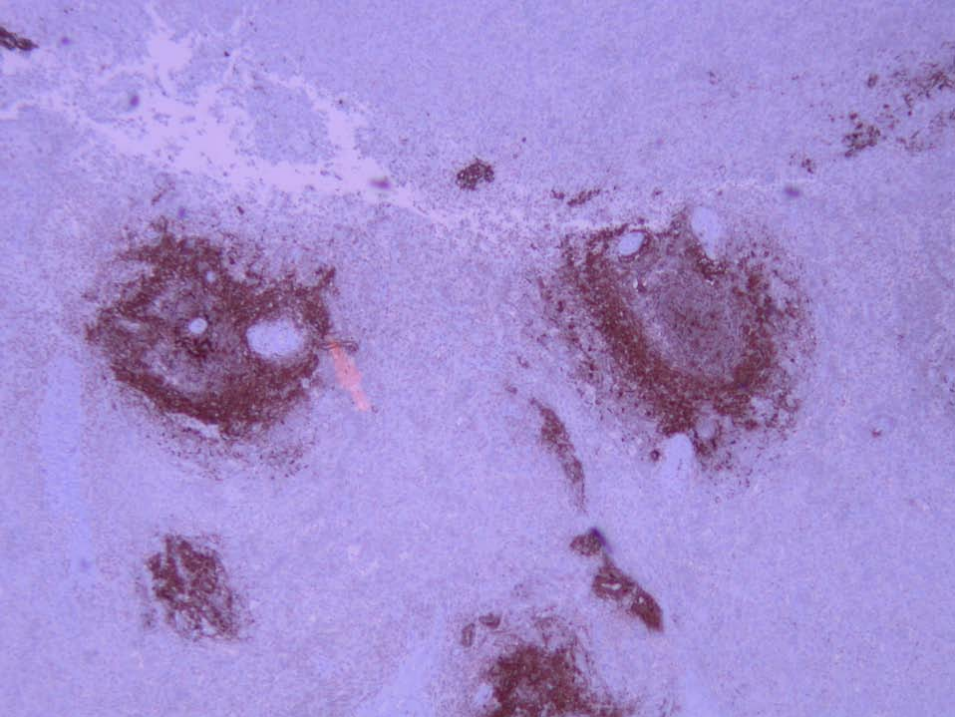
**Peri-vascular amyloid**. Positive peri-vascular amyloid A immunoperoxidase staining at 40 × magnification.

When the gross spleen was sectioned along its short axis in the operating room (Figure 3) no abscess was found. Microbiological examination revealed few polymorphonuclear leukocytes (PMNs), many red blood cells, and rarely *S. aureus *bacteria. A pathological examination revealed very few PMNs and none of the acute inflammation expected in cases of splenic infarction. The splenic red pulp was almost totally replaced by plasma cells. Staining for amyloid was strikingly positive (Figure 4). Characteristic talc crystals were observed inside the splenic parenchyma (Figure 5).

## Discussion

Amyloid, which appears as pink, amorphous, intercellular material in tissue sections stained with hematoxylin and eosin, can be deposited in any tissue, and may be localized or widespread in its deposition. The most common sites are liver, spleen, adrenal, and kidney, where amyloid is frequently deposited around vascular structures. Amyloidosis is subclassified by the type of proteins that make up the amyloid fibrils. The basic structure of all amyloid types is a β-pleated sheet of fibrils 7.5 to 10 nm wide. Numerous methods can demonstrate the presence of amyloid, but the most specific is Congo Red stain, followed by microscopic examination with polarizing lenses that show a characteristic bright green birefringence. Immunoperoxidase staining is then performed to detect acquired systemic AA amyloidosis [6].

The most common type of amyloidosis in the USA is immunological. This type is composed of light chains (Bence-Jones proteins) secreted by plasma cells of multiple myeloma [6,7]. Amyloidosis may also be secondary to chronic inflammatory conditions such as autoimmune diseases, chronic infections, and some neoplasms (for example, Hodgkin's lymphoma and renal cell carcinoma) [1]. The amyloid fibrils in inflammatory conditions are composed of serum amyloid-associated protein. The heredofamilial type of amyloidosis is an autosomal dominant condition [8].

Pre-operatively, our patient was presumed to have splenic infarct or abscess as the cause of splenic rupture because up to 51% of patients with IE suffer emboli to major organs [9]. During surgery, we questioned that diagnosis when no abscess was seen in the spleen, and the cross-sectioned tissue appeared grossly homogeneous.

Pathological evaluation was consistent with acquired systemic AA amyloidosis, which was previously called secondary amyloid because it was seen secondary to inflammation. This type of amyloidosis is a rare systemic condition that can occur in the context of chronic inflammation in which there is protracted breakdown of cells. This condition is seen most commonly in rheumatoid arthritis, ankylosing spondylitis, inflammatory bowel disease, and the drug use method referred to as 'skin popping', as in our patient [10]. Skin popping is the injection of narcotics, commonly heroin, just under the skin. Black tar heroin is used on the west coast of the USA and is considerably 'dirtier' than its east coast heroin counterpart. Users tend to have a high incidence of abscess formation, IE, and possibly reactive systemic amyloidosis, as in our patient. The cytokine release during chronic bouts of inflammation is thought to lead to increased production of AA protein in the liver; this protein is then released into the blood stream and deposited in small blood vessels throughout the body. This protein deposition leads to vessels that are delicate and easily disrupted.

Patients who are hospitalized rarely experience ASR. However, septic emboli are common in patients with IE who are hospitalized, occurring in up to 51% [9]. In left-sided endocarditis, these emboli can travel to the spleen and lead to infarction and splenic rupture. Our patient was hospitalized for IE, but pathological evaluation of the spleen revealed no evidence of bacterial embolization, infarction or abscess; therefore, the ASR most likely resulted from systemic AA amyloidosis.

## Conclusion

Patients with a history of 'skin popping', especially with black tar heroin, are at risk for AA amyloidosis. In patients with chronic inflammatory conditions who develop an acute onset of abdominal pain, the possibility of splenic rupture should be considered, even if no history of trauma can be elicited.

## Consent

Written informed consent for publication could not be obtained because the patient is now deceased and we were unable to contact a next of kin despite all reasonable attempts. Every effort has been made to protect the identity of the patient and there is no reason to believe that the patient or their family would object to publication.

## Competing interests

The authors declare that they have no competing interests.

## Authors' contributions

GRR contributed to the surgical care of our patient, the literature review and manuscript preparation and revision. AYL contributed to the literature review and manuscript revision. KR contributed to the surgical care for our patient and manuscript revision. BV contributed to the surgical care of our patient and direction of manuscript preparation. DKH performed the histological analysis of our patient's spleen, contributed the pathology discussion in the manuscript and prepared the images. MMK contributed to the surgical care of our patient, and manuscript preparation and revision. FJR contributed to the medical care of our patient and oversaw manuscript preparation and revision. All authors read and approved the final manuscript.
